# *Mycobacterium tuberculosis* Rv3586 (DacA) Is a Diadenylate Cyclase That Converts ATP or ADP into c-di-AMP

**DOI:** 10.1371/journal.pone.0035206

**Published:** 2012-04-17

**Authors:** Yinlan Bai, Jun Yang, Xin Zhou, Xinxin Ding, Leslie E. Eisele, Guangchun Bai

**Affiliations:** 1 Center for Immunology and Microbial Disease, Albany Medical College, Albany, New York, United States of America; 2 Wadsworth Center, New York State Department of Health, Albany, New York, United States of America; 3 School of Public Health, State University of New York at Albany, Albany, New York, United States of America; Queen Mary University of London, United Kingdom

## Abstract

Cyclic diguanosine monophosphate (c-di-GMP) and cyclic diadenosine monophosphate (c-di-AMP) are recently identified signaling molecules. c-di-GMP has been shown to play important roles in bacterial pathogenesis, whereas information about c-di-AMP remains very limited. *Mycobacterium tuberculosis* Rv3586 (DacA), which is an ortholog of *Bacillus subtilis* DisA, is a putative diadenylate cyclase. In this study, we determined the enzymatic activity of DacA *in vitro* using high-performance liquid chromatography (HPLC), mass spectrometry (MS) and thin layer chromatography (TLC). Our results showed that DacA was mainly a diadenylate cyclase, which resembles DisA. In addition, DacA also exhibited residual ATPase and ADPase *in vitro*. Among the potential substrates tested, DacA was able to utilize both ATP and ADP, but not AMP, pApA, c-di-AMP or GTP. By using gel filtration and analytical ultracentrifugation, we further demonstrated that DacA existed as an octamer, with the N-terminal domain contributing to tetramerization and the C-terminal domain providing additional dimerization. Both the N-terminal and the C-terminal domains were essential for the DacA's enzymatically active conformation. The diadenylate cyclase activity of DacA was dependent on divalent metal ions such as Mg^2+^, Mn^2+^ or Co^2+^. DacA was more active at a basic pH rather than at an acidic pH. The conserved RHR motif in DacA was essential for interacting with ATP, and mutation of this motif to AAA completely abolished DacA's diadenylate cyclase activity. These results provide the molecular basis for designating DacA as a diadenylate cyclase. Our future studies will explore the biological function of this enzyme in *M. tuberculosis*.

## Introduction

Tuberculosis (TB) remains a global epidemic, with one-third of the world's population infected and approximately 9 million new active cases annually [1]. The TB epidemic is exacerbated by a synergy with human immunodeficiency virus (HIV) and steadily increasing rates of drug resistance [2], [3]. The efficacy of the only vaccine strain, *Mycobacterium bovis* BCG, varies from 0 to 80% in preventing pulmonary TB [4]. Therefore, new strategies for TB therapy and novel vaccines for eradication of the infection are urgently needed. A better understanding of the signaling mechanism of *Mycobacterium tuberculosis* could facilitate these goals.

Several cyclic nucleotides have been shown to play important roles in bacterial gene regulation and pathogenesis. These nucleotides include cyclic adenosine monophosphate (cAMP), cyclic guanosine monophosphate (cGMP), cyclic diguanosine monophosphate (c-di-GMP) and cyclic diadenosine monophosphate (c-di-AMP) [5]. cAMP has been well studied in a large number of bacteria. This signaling molecule regulates gene expression in response to diverse environmental conditions [6]. In *M. tuberculosis*, at least 15 adenylate cyclases have been identified [7], [8], [9], [10]. The *M. tuberculosis* complex bacteria are able to secrete significant amount of cAMP into infected macrophages [11], [12], [13]; this event may play a role during infection [11]. c-di-GMP is another important second messenger that is widespread in bacteria. It is synthesized from two GTP molecules by diguanylate cyclase and can be converted into pGpG or GMP by various phosphodiesterases [14], [15], [16]. c-di-GMP has been known to play a role in the regulation of the biological cascades relevant to bacterial pathogenesis [14], [15], [17], [18], [19], [20].

c-di-AMP has recently been recognized as a signaling molecule. This nucleotide is synthesized from ATP by diadenylate cyclase and is linearized to pApA by c-di-AMP phosphodiesterase. The diadenylate cyclase has been identified in *Bacillus subtilis*, *Thermotoga maritima* [21], [22], *Listeria monocytogenes* [23], *Staphylococcus aureus* [24] and *Streptococcus pyogenes* [25]. In *B. subtilis*, the cyclase is named as DisA (or YacK) for DNA integrity scanning protein A, which is involved in cell-cycle checkpoints. DisA forms a large octamer, and each monomer consists of a nucleotide-binding domain and two DNA binding domains [22]. DisA converts ATP into c-di-AMP, but does not utilize GTP as a substrate [22]. In *B. subtilis*, bacterial c-di-AMP levels are reduced in response to DNA damage, which results in a delay of sporulation. This phenotype can be corrected by supplementation of exogenous c-di-AMP [26]. A diadenylate cyclase and a c-di-AMP phosphodiesterase have been characterized in *S. aureus*. Deletion of the phosphodiesterase in this pathogen results in smaller bacterial size and alteration in biofilm formation [24]. *B. subtilis* YybT protein hydrolyzes c-di-AMP and c-di-GMP into linear pApA and pGpG, respectively [16]. Bacterial c-di-AMP also modulates host immune responses. It has been reported that c-di-AMP secreted by *L. monocytogenes* represents a putative secondary signaling molecule that triggers a cytosolic pathway of innate immunity [23]. This response is likely mediated by Sting, a host transmembrane protein [27], [28]. In addition, c-di-AMP has been recognized as an effective immunoadjuvant that promotes strong Th1/Th2/Th17 responses [29].

The *M. tuberculosis* Rv3586 protein is a putative DisA ortholog. The two motifs (DGA and RHR) that are conserved in DisA proteins of *T. maritima* and *B. subtilis* are also conserved in the Rv3586 [22]. The significance of c-di-AMP in other pathogens implicates that characterization of the Rv3586 might provide new insights into the biology of signal transduction in TB pathogenesis. However, Rv3586 and c-di-AMP in mycobacteria have not been experimentally explored. In this study, we show that the Rv3586 protein is functional as a diadenylate cyclase (Dac). Therefore, we designated this protein DacA and its encoding gene *dacA*, based on our results and the published records of other bacteria [23], [24]. This is the first report describing the existence of a functional diadenylate cyclase in *M. tuberculosis*.

## Materials and Methods

### Ethics statement

This study was carried out in strict accordance with the recommendations in the Guide for the Care and Use of Laboratory Animals of the National Institutes of Health. The protocol was approved by the Institutional Animal Care and Use Committee of Albany Medical College (Permit Number: 11-02005). All efforts were made to minimize suffering.

### Bacterial strains and culture conditions

*M. tuberculosis* H37Rv was grown in Difco Middlebrook 7H9 medium (BD) supplemented with 0.5% glycerol, 10% oleic acid-albumin-dextrose-catalase (OADC), 0.05% Tween-80, as previously described [30]. Bacteria were grown to late log phase to isolate genomic DNA. *E. coli* DH5α and BL21(DE3) were grown in Luria-Bertani broth or on Luria-Bertani agar plates. Kanamycin at 25 µg/ml was added for all recombinant strains. All cultures were grown at 37°C.

### Protein expression and purification

The *M. tuberculosis dacA* open reading frame (ORF) and the truncated DNA fragments encoding the first 287 aa (DacA_1–287_) and the last 219 aa (DacA_140–358_) of DacA were PCR amplified using the primers listed in Table 1. The *M. tuberculosis* H37Rv genomic DNA was used as a template. The PCR products for DacA, DacA_1–287_ and DacA_140–358_ were cloned into pET28a(+) vector (Novagen) between NcoI and HindIII sites to generate pMBC1218, pGB067 and pGB068, respectively. These plasmids were sequence verified and maintained in *E. coli* BL21(DE3).

**Table 1:** Primers used for protein expression in this study^a^.

Protein	Primer	Oligo sequence (5′ to 3′)
DacA	KM2948	GCGCCATGGAGCACGCTGTGACTCGTCCGACC
	KM2949	GCGAAGCTTTTGATCGCTGATGGTCGATTCC
DacA_1–287_	JY078	GCGAAGCTTCGAATCCTGCGCTTCCGTGG
DacA_140–358_	JY077	GCGCCATGGTATTGACCGACTCGGCAACC
DacA_DG_	JY178	GAGCTGTGCAAGATG**GCCGCC**GCCGTGGTGCTGTC
	JY179	GACAGCACCACGGC**GGCGGC**CATCTTGCACAGCTC
DacA_DGA_	JY199	TACGCGAGCTGTGCAAGATG**GCAGCATTA**GTGGTGCTGTCCACCGACGG
	JY200	CCGTCGGTGGACAGCACCAC**TAATGCTGC**CATCTTGCACAGCTCGCGTA
DacA_RHR_	JY201	CCACCGACGAATCGGGGACC**GCTGCTGCA**TCGGCCGAGCGGGCCGCGAT
	JY202	ATCGCGGCCCGCTCGGCCGA**TGCAGCAGC**GGTCCCCGATTCGTCGGTGG
DacA_G73A_	JY218	GCTGTGCAAGATGGAC**GCC**GCCGTGGTGCTGTCC
	JY219	GGACAGCACCACGGC**GGC**GTCCATCTTGCACAGC

a , Primer JY078 was used in combination with KM2948, and primer JY077 was used in combination with KM2949. The mutations for the respective amino acids in primers JY178 to JY219 are indicated in bold.

Mutations of DGA (aa 72–74) and RHR (aa 105–107) motifs in DacA were generated using SOEing PCR similarly as we reported [31]. Primers KM2948, JY199, JY200 and KM2949 (Table 1) were used to replace DGA with AAL (DacA_DGA_); primers KM2948, JY178, JY179 and KM2949 (Table 1) were used to replace DGA with AAA (DacA_DG_); and primers KM2948, JY201, JY202 and KM2949 (Table 1) were used to substitute RHR with AAA (DacA_RHR_). Since expression of both DacA_DGA_ and DacA_DG_ was only detected in inclusion bodies, a point mutation (DacA_G73A_) in DGA motif of DacA was generated using primers KM2948, JY218, JY219 and KM2949 (Table 1). All the final PCR products were digested with NcoI and HindIII, cloned into pET28a(+) vector as described above, and verified by sequencing. The plasmids for DacA_DGA_, DacA_RHR_, DacA_DG_ and DacA_G73A_ were designated as pGB125, pGB126, pGB137 and pGB141, respectively. These plasmids were maintained in BL21(DE3) for protein expression.

The expression of the proteins was induced with 0.05 mM isopropyl β-D-1-thiogalactopyranoside (IPTG) for 3 h at room temperature, except that DacA_RHR_ was expressed at 16°C. The C-His-tagged recombinant proteins were purified using a Ni-NTA resin (Qiagen) with buffers as we previously reported [32], [33].

### Gel filtration

Size-exclusion chromatography experiments were performed with a Superdex 200 column (10×300 mm) connected to a Gradiphrac Automatic Sampler (Amersham Biosciences). The column was equilibrated and eluted with the running buffer (10% glycerol in PBS at pH 7.4) at a constant flow rate of 0.5 ml/min. Molecular mass of the proteins was determined by using Gel Filtration Standard (Bio-Rad) per the instruction in Gel Filtration Principles and Methods (GE Healthcare). The protein concentrations were then determined using BCA Protein Assay Kit (Thermo Scientific). The purified proteins were stored in aliquots at −80°C.

### Analytical ultracentrifugation

Sedimentation velocity studies were performed using a Beckman Coulter XL-1 analytical ultracentrifuge and an An-60 Ti rotor at 4°C as described earlier [34], except that the proteins were analyzed in a buffer containing 10% glycerol in PBS. DacA and DacA_1–287_ were analyzed at 40 000 rpm, and DacA_140–358_ was analyzed at 50 000 rpm. The volume of protein samples was 400 µl, and the reference buffer volume was 420 µl. The viscosity and the density of the buffer and the partial specific volume of the proteins were determined using the SEDNTERP software. The data were analyzed by the *c*(*s*) and the *c*(*M*) methods found in SEDFIT, a program developed by Schuck [35]. Because the molecular distribution consisted of a single major peak, the *c*(*M*) method was used to estimate the molecular mass of the main species [36].

### High-performance liquid chromatography (HPLC)

Determination of DacA's enzymatic activities using HPLC was performed as reported [22], [37] with minor modification. Briefly, reaction mixtures (10 µl) contained 40 mM Tris-HCl (pH 7.5), 10 mM MgCl_2_, 100 mM NaCl and nucleotide as specified. The reaction was initiated by adding 2.5 µM protein and was incubated at 37°C for 1 h. The reaction was then terminated by adding 1 µl of 0.5 M EDTA, followed by a 1∶5 dilution with water. Finally, 20 µl of each sample was injected and separated by reverse-phase HPLC with a C18 column (250×4.6 mm, Vydac) using a Waters 625 LC system equipped with a 996 Photodiode Array Detector and a 717 Autosampler (Waters). Samples were eluted using the same buffers and program as reported [38]. Nucleotides were monitored at 254 nm. c-di-AMP and pApA standards were purchased from BioLog. ATP, GTP, ADP and AMP were purchased from Sigma.

### Mass spectrometry (MS)

The reaction mixture (10 µl) as described for HPLC analysis was diluted 50 times, and 10 µl was analyzed using an LC/UV/MS system consisting of an Agilent 1200 separation module, an Agilent 1260 photodiode array detector (Agilent) and an ABI 4000 Q-Trap mass spectrometer (Applied Biosystems). The chromatographic separation of products was achieved on a 5-µm Gemini C18 (150×2.0 mm) column (Phenomenex). The mobile phase consisted of solvent A (10 mM ammonium acetate in water) and solvent B (100% acetonitrile). The samples were eluted, at a flow rate of 0.2 ml/min, with 100% A for 5 min, followed by linear increases from 0% B to 100% B between 5 and 10 min, and then 100% B for a further 2.5 min. The enhanced full mass scan (EMS) was conducted at a mass range of 100 to 1000 amu with a scan rate of 1000 Da/s, and the mass spectrometer was operated in a negative ion mode with an electrospray ionization source. The parameters for the chamber were as follows: curtain gas, 50 psi; heated nebulizer temperature 400°C; ion spray voltage, −4500 V; gas 1, 50 psi; gas 2, 50 psi, declustering potential, −50 V; collision energy −10 eV; CAD gas, medium.

### Hydrolysis of nucleotides and thin layer chromatography (TLC)

For TLC samples, reaction mixture (10 µl) contained 40 mM Tris-HCl (pH 7.5), 10 mM MgCl_2_, 100 mM NaCl, 100 µM unlabeled ATP and 0.01 µCi/µl of [α-^33^P]ATP (MP Biomedicals). The reaction was initiated by adding proteins as specified and incubated at 37°C for various time periods as indicated in Results. An aliquot of 2.5 µl was removed at each time point and immediately mixed with an equal volume of 0.5 M EDTA. One microliter of this mixture was finally spotted onto a pre-coated polyethyleneimine-cellulose plate (Sigma) and was separated with a solvent containing 1∶1.5 (v/v) saturated (NH_4_) _2_SO_4_ and 1.5 M KH_2_PO_4_ (pH 3.6) for 1 h. The dried plate was exposed on a phosphor screen, scanned with a Storm 860 PhosphorImager (Molecular Dynamics), and analyzed using ImageQuant software (Molecular Dynamics). For nucleotide standards, one microliter from a 5-mM stock of each unlabeled nucleotide was spotted onto a TLC plate and separated using the same solvent. Image was taken under 254 nm UV light.

### Metal ion and pH dependence

The assay conditions used for metal ion screening were: 40 mM Tris-HCl (pH 7.5), 100 mM NaCl, 2 mM [metal^2+^], 2.5 µM DacA and 2 mM ATP or 0.5 mM ADP. For pH analysis, reactions consisted of 100 mM NaCl, 10 mM MgCl_2_, 2.5 µM DacA, 2 mM ATP or 0.5 mM ADP, and 40 mM Tris-HCl at pH 6.0, 6.5, 7.0, 7.5 and 8.0, respectively. Reactions were incubated for 1 h at 37°C, terminated by adding 1 µl of 0.5 M EDTA and analyzed by HPLC. The peak areas of c-di-AMP were compared and presented as arbitrary units.

### Preparation of polyclonal antibody against DacA

Five female BALB/c mice (Taconic) were immunized subcutaneously with 50 µg of purified DacA emulsified 1∶1 with Alum (Thermo Scientific) in 100 µl and boosted twice biweekly with the same amount of the protein and the adjuvant. The specificity of serum was analyzed by Western blot with the purified DacA protein. The protein was blotted onto polyvinylidene fluoride (PVDF) membranes and sequentially probed with the anti-DacA antibody we generated and with a peroxidase-conjugated goat anti-mouse IgG secondary antibody (Thermo Scientific). Peroxidase detection was carried out with the ECL Western blotting detection reagents and analysis system (Thermo Scientific).

### Cross-linking of protein

Cross-linking of purified DacA, DacA_1–287_ and DacA_140–358_ was performed as described earlier [32], [39] with slight modification. Each protein was diluted in cross-linking buffer (50 mM sodium phosphate, pH 7.4, 20% glycerol, 5 mM MgCl_2_) to 1.5 µM, and was then incubated with glutaraldehyde at a final concentration of 35 mM for 1 h at room temperature. The reaction was quenched by the addition of SDS-PAGE sample buffer, and a portion of each protein sample was separated on a 10% SDS-PAGE gel. The protein was transferred onto a PVDF membrane and visualized after Western blot with the anti-DacA antibody.

### ATP binding assay

The ATP binding with DacA, DacA_1–287_, DacA_140–358_, DacA_G73A_ and DacA_RHR_ was analyzed using a gel mobility shift assay. Briefly, the reaction mixture (10 µl) contains 40 mM Tris-HCl (pH 7.5), 100 mM NaCl and 2 µg of protein either in the presence or in the absence of 10 mM ATP. The reaction mixtures were prepared and incubated on ice for 10 min. Samples were then loaded onto a 6% polyacrylamide native gel and separated with 0.5× TBE at 4°C. Proteins were visualized by staining with 0.25% Coomassie Brilliant Blue G250.

### Bioinformatics and structural modeling

The amino acid sequence of *M. tuberculosis* DacA was aligned with that of *T. maritima* DisA using the Clustal W method of MegAlign software (DNAStar). For structural modeling, the crystal structure of *T. maritima* DisA was obtained from the entry 3C23 of Protein Data Bank (PDB) [22] using the Cn3D program of the National Center for Biotechnology Information (NCBI, version 4.3).

## Results

### Oligomerization of DacA

**Figure 1:**
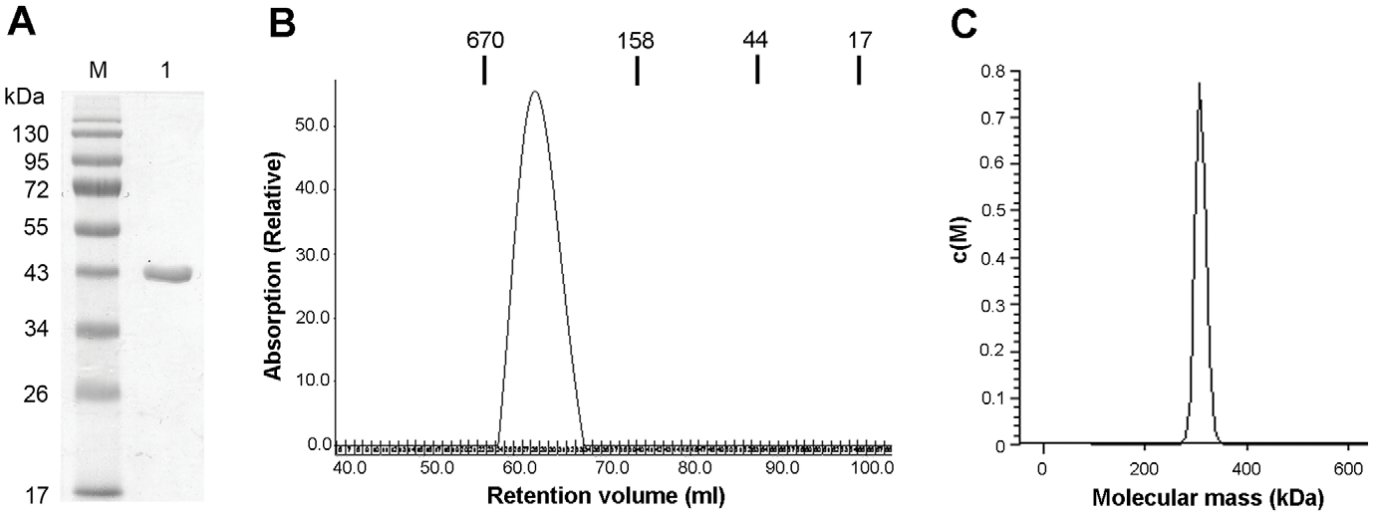
Purification and oligomerization of DacA. (A) SDS-PAGE of purified DacA. Lane M, EZ-Run Pre-stained Rec Protein Ladder (Fischer Scientific); lane 1, purified DacA. (B) Gel filtration chromatograph of DacA. The molecular weights (in kDa) and the retention volumes of the standards are indicated on the top. (C) Analytical ultracentrifugation of DacA. Molecular mass of DacA was estimated using the *c*(*M*) method.

We expressed and purified the C-terminal His-tagged DacA in *E. coli* to high homogeneity. The apparent molecular weight of this protein was about 43 kDa on a SDS-PAGE gel (Fig. 1A), which is consistent with the calculated molecular weight (41.4 kDa). Gel filtration analysis with the purified protein revealed that DacA formed a highly stable octamer with an estimated molecular mass of 330 kDa (Fig. 1B), which is equivalent to the calculated molecular weight of octamerized DacA (331.2 kDa). In addition, sedimentation velocity study of DacA demonstrated a molecular mass of ∼307 kDa (Fig. 1C), supporting the notion that DacA exists as an octamer.

### DacA is an ortholog of DisA as a diadenylate cyclase

**Figure 2:**
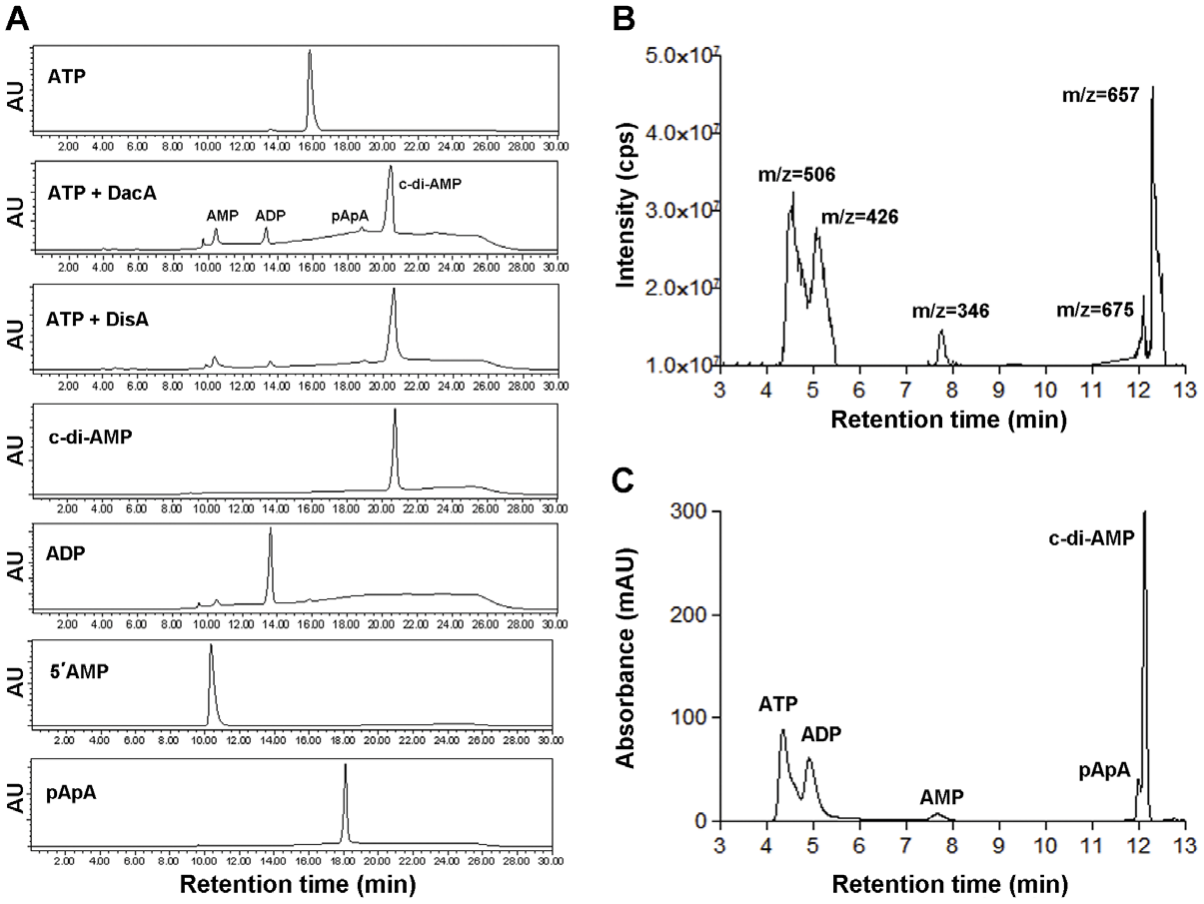
Determination of DacA's activities using HPLC and LC-MS. (A) Analysis of the products from reaction of ATP with DacA using HPLC. Reaction of ATP with DisA was included as a positive control. The reactions were carried out as described in the Methods. ATP, c-di-AMP, ADP, AMP and pApA standards were also analyzed under the same conditions. (B and C) LC/UV/MS profiles of the products formed by DacA with ATP. The products were detected by monitoring EMS at mass range from 100 to 1000 amu (B) or monitored by UV absorption at 254 nm (C).

Amino acid sequence alignment revealed that *M. tuberculosis* DacA shares 42% identity with *B. subtilis* DisA. The two putative motifs of DisA, DGA and RHR, are conserved in DacA. These analyses suggest that DacA is a putative diadenylate cyclase. We first performed an enzymatic analysis of DacA with ATP by using HPLC. The results showed that DacA converted ATP into a major product with the same retention time as that of the product of DisA, which is c-di-AMP (Fig. 2A). This result was also supported by HPLC of the c-di-AMP standard (Fig. 2A). Interestingly, we noticed that three minor peaks were also present in the reaction of DacA with ATP (Fig. 2A), but not in a control reaction that was carried out in the absence of ATP (not shown), suggesting that DacA may have activities other than that of a diadenylate cyclase. The identities of the DacA-catalyzed products were detected using LC/UV/MS. The total ion chromatogram (TIC) of EMS analysis displayed four products derived from ATP (Fig. 2B). Extracted ion chromatogram (EIC) processing of the EMS dataset for *m/z* 346, 426, 675 and 657 revealed the identities of the products, which are consistent with the molecular ion of AMP, ADP, pApA and c-di-AMP, respectively. The identities of the products were confirmed based on coelution with authentic standards under the same LC conditions (Fig. 2C) or by comparing the retention times with those of purified standards (Fig. 2A).

**Figure 3:**
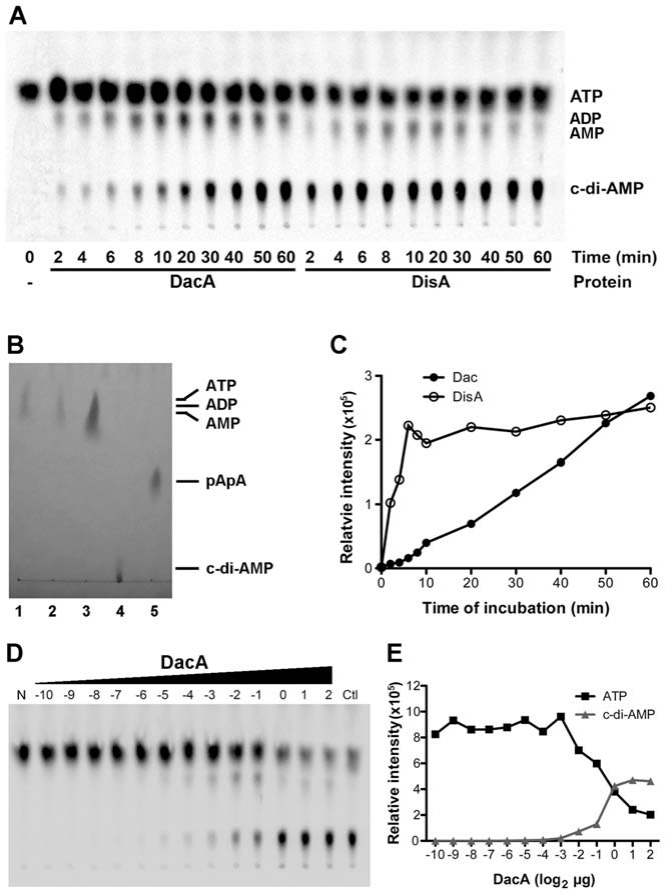
Determination of DacA's activities using TLC. (A) Separation of nucleotides generated from [α-^33^P]ATP by DacA and DisA. The positions of ATP, ADP, AMP and c-di-AMP are indicated based on the *R_f_* of each standard analyzed in panel B under the same conditions. (B) Separation of nucleotide standards using TLC. Spots 1–5 are ATP, ADP, AMP, c-di-AMP and pApA, respectively. (C) Quantitation of c-di-AMP production. The relative intensity of c-di-AMP generated by DacA or DisA at various time points as in panel A was analyzed using the ImageQuant software. Data shown are representative of two repeat experiments. (D) Production of c-di-AMP with various concentrations of DacA at 30 min of incubation. Reactions contain 2-fold serial diluted DacA protein as indicated on the top of the TLC graph (in log_2_ µg). “N” indicates a control with no protein, and “Ctl” contains 1 µg DisA as a positive control. “0” equals 1 µg of protein. (E) Quantitation of ATP depletion and c-di-AMP production by DacA from panel D. Data shown are representative of two repeat experiments.

We further analyzed [α-^33^P]-labeled nucleotides in the catalytic reaction by using TLC (Fig. 3A), in which the order of migration away from the origin (*R_f_*) was c-di-AMP, AMP, ADP and ATP, as determined through comparisons with unlabeled standards (Fig. 3B). By mixing DacA with [α-^33^P]ATP, c-di-AMP was formed in a time-dependent manner (Fig. 3A). The reaction rate of DacA was slower than DisA (Fig. 3A,C). At ∼50 min, the production of c-di-AMP catalyzed by DacA was equivalent to that by DisA, which was saturated at ∼6 min (Fig. 3A, C). This result indicates that DacA is ∼5 to 10-fold less active in synthesis of c-di-AMP than that of the DisA control.

ADP and AMP were also detected using TLC from the reaction catalyzed by DacA, while AMP was detected in the reaction with DisA (Fig. 3A), similar to the HPLC data (Fig. 2A). These nucleotides are likely secondary products produced by DacA or DisA. Noticeably, the ADP in DacA reactions and the AMP in DisA reactions were more abundant at 10 to 40 min than those at 50 to 60 min (Fig. 3A), indicating that these nucleotides may also be used as substrates by the respective diadenylate cyclase. In the presence of increasing amounts of DacA in the reaction, the c-di-AMP production, but not the yield of the secondary products, was clearly dependent on the enzyme concentration (Fig. 3D, E).

In addition to ATP, we also determined the enzymatic activity of DacA with GTP as a potential substrate using HPLC. We did not find any peak other than GTP, indicating that the activity of DacA is specific to ATP, but not GTP (data not shown).

Taken together, these results indicate that the major activity of DacA is as a diadenylate cyclase, while the minor activities may include those of ATPase and ADPase.

### DacA utilizes both ATP and ADP as substrates

**Figure 4:**
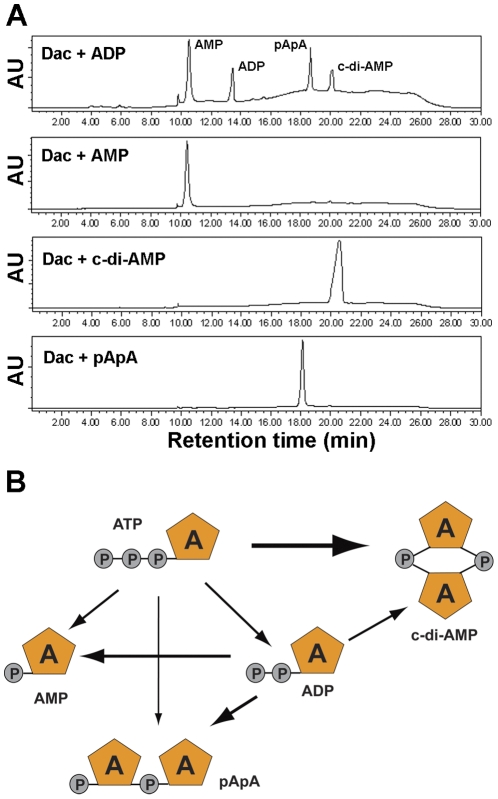
Catalytic activities of DacA with different nucleotides. (A) Reaction of DacA with ADP, AMP, c-di-AMP or pApA. Samples were separated by HPLC. The peaks in “DacA+ADP” are labeled according to the retention time of each standard as shown in Fig. 2A. (B) Reactions catalyzed by DacA using ATP as a substrate, based on the results shown in Fig. 2 and Fig. 4A. “A” stands for adenosine, and “P” stands for phosphate. The thickness of arrows denotes priority of reaction, and the thickest arrow shows the major catalytic reaction.

It has been well known that c-di-GMP can be hydrolyzed into pGpG and GMP by various phosphodiesterases. Many diguanylate cyclases also have a c-di-GMP phosphodiesterase domain. In this study, we noticed that in the reaction with ATP and DacA, AMP, ADP, pApA and c-di-AMP were all present as products (Fig. 2). These products might be converted directly from either ATP or c-di-AMP. Therefore, we determined the enzymatic activity of DacA with ADP, AMP, c-di-AMP and pApA, respectively. Interestingly, DacA converted ADP into AMP, pApA and c-di-AMP, although the yield of c-di-AMP from ADP was much lower compared with that from ATP (Fig. 4A). In contrast, no additional product was detected in reactions of DacA with AMP, c-di-AMP or pApA (Fig. 4A). These results suggest that DacA does not have c-di-AMP phosphodiesterase activity. Based on our results, we have proposed a model of DacA's activities (Fig. 4B), which shows that DacA catalyzes the conversion of both ATP and ADP into c-di-AMP. Meanwhile, DacA also produces ADP, AMP and pApA.

### Effect of metal ion and pH on DacA's activity

**Figure 5:**
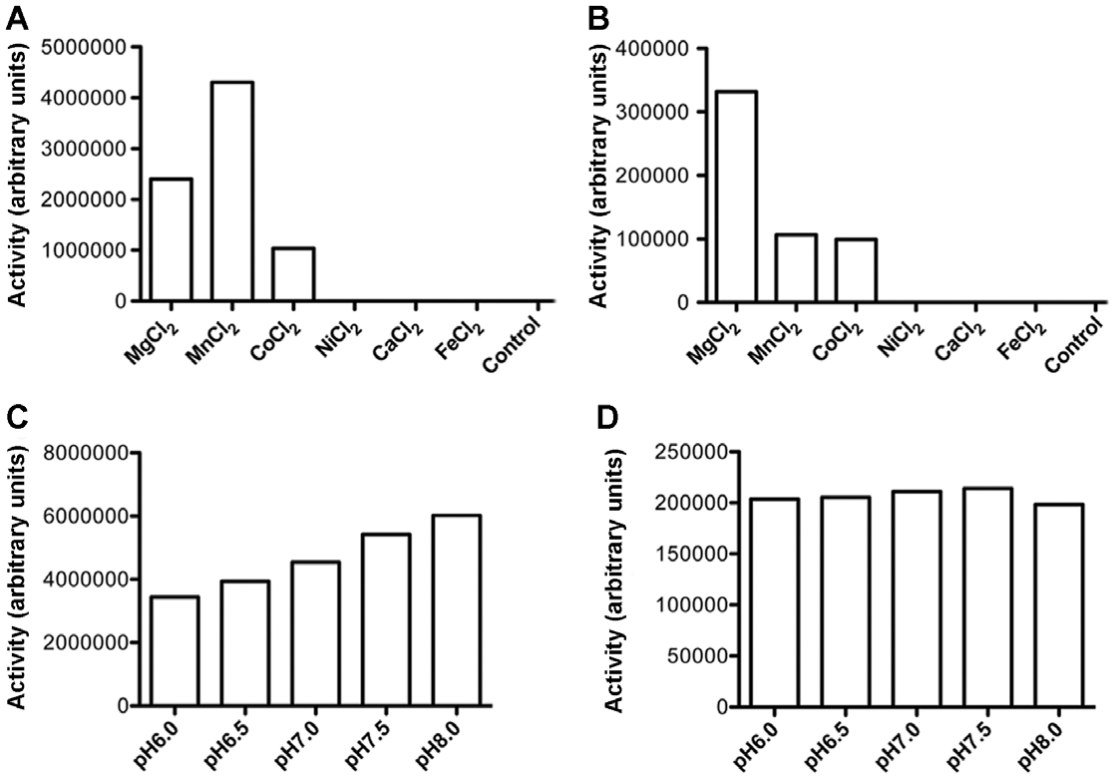
Effect of divalent metal ions and pH on DacA's activities. (A and B) Effect of metal ions on c-di-AMP production catalyzed by DacA in the presence of 2 mM ATP (A) or 0.5 mM ADP (B). (C and D) Effect of pH on c-di-AMP production catalyzed by DacA in the presence of 2 mM ATP (C) or 0.5 mM ADP (D). Note that less ADP was used in the reactions compared with ATP, and thus the arbitrary units between reactions with ATP and ADP are not directly comparable.

The effect of metal ion on the diadenylate cyclase activity of DacA was analyzed using six divalent ions: Mg^2+^, Mn^2+^, Co^2+^, Ni^2+^, Ca^2+^ and Fe^2+^. When ATP was provided, the production of c-di-AMP was detected in the reactions with Mg^2+^, Mn^2+^ or Co^2+^, but not with the other ions. Under our testing conditions, DacA preferred Mn^2+^>Mg^2+^>Co^2+^ as co-factors (Fig. 5A). When ADP was utilized, the diadenylate cyclase activity was also detected in the presence of Mg^2+^, Mn^2+^ or Co^2+^, but not with the other ions (Fig. 5B). More c-di-AMP was converted from ADP in the presence of Mg^2+^ than in the presence of Mn^2+^ or Co^2+^ (Fig. 5B). As controls, DacA did not show any enzymatic activity in the absence of any divalent metal ion when either ATP or ADP was provided (Fig. 5A, B).

It is well known that environmental pH may affect the structures and activities of enzymes. In this study, we determined the diadenylate cyclase activity of DacA at pH 6.0, 6.5, 7.0, 7.5 and 8.0, respectively, using either ATP or ADP as a substrate. In the presence of ATP, DacA produced more c-di-AMP at a basic pH than at an acidic pH; the activity was increased by almost two fold at pH 8.0, compared to that at pH 6.0 (Fig. 5C). However, when ADP was provided, no difference was detected by changing pH from 6.0 to 8.0 (Fig. 5D).

### Both the N-terminal and the C-terminal domains are required for DacA's activity

**Figure 6:**
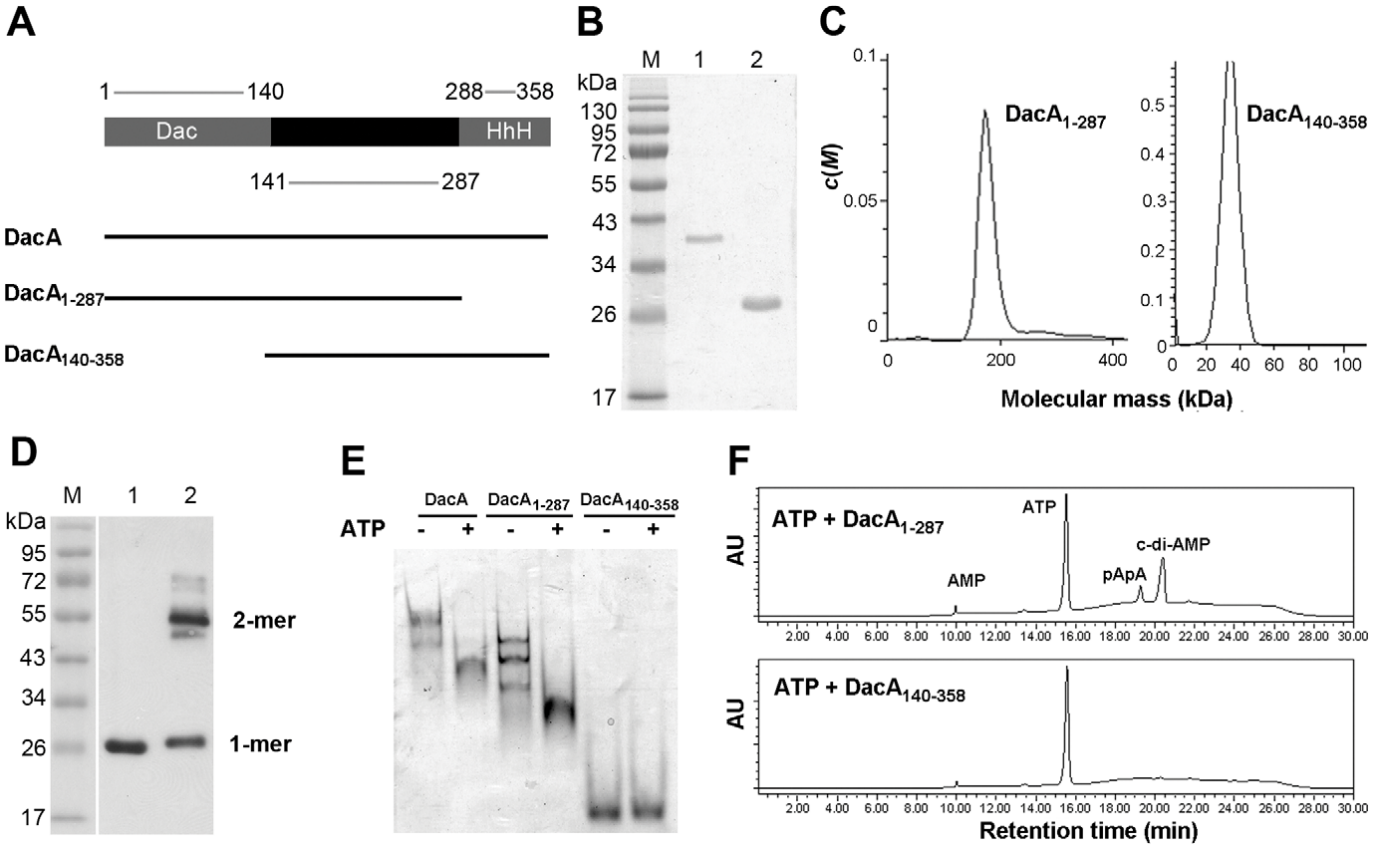
Function of the N-terminal and the C-terminal domains of DacA in oligomerization and enzymatic activity. (A) Schematic representation of the primary structures of DacA, DacA_1–287_ and DacA_140–358_, as indicated with black lines. DacA_1–287_ lacks the C-terminal HhH domain, while DacA_140–358_ lacks the N-terminal Dac domain. (B) SDS-PAGE of purified DacA_1–287_ and DacA_140–358_. Lane M, MW marker; lanes 1 and 2 are purified DacA_1–287_ and DacA_140–358_, respectively. (C) Analytical ultracentrifugation of DacA_1–287_ and DacA_140–358_. (D) Cross-linking of DacA_140–358_ with glutaraldehyde. Lane M, MW marker; lane 1, untreated DacA_140–358_; and lane 2, glutaraldehyde-treated DacA_140–358_. Lanes 1 and 2 were analyzed using Western blot with the anti-DacA antibody. (E) ATP binding by DacA, DacA_1–287_ and DacA_140–358_. Proteins, either in the presence (+) or absence (−) of ATP, were separated by electrophoresis with a native gel and stained with Coomassie Brilliant Blue. (F) Enzymatic activity of 10 µM DacA_1–287_ and DacA_140–358_ analyzed using HPLC.

Based on the sequence analysis using the Web CD Search Tool (http://www.ncbi.nlm.nih.gov/Structure/bwrpsb/bwrpsb.cgi) and the sequence alignment with DisA [22], DacA possesses three domains (Fig. 6A). To determine which domain is important for DacA's activity, we generated two truncated forms of DacA, DacA_1–287_ and DacA_140–358_, which lacks the C-terminal domain and the N-terminal domain, respectively. We then determined the oligomerization of these proteins and their enzymatic activity as a diadenylate cyclase. The apparent molecular weights of DacA_1–287_ and DacA_140–358_ were about 37 kDa and 27 kDa, respectively, on a SDS-PAGE gel (Fig. 6B), which are consistent with their calculated molecular weights (33.8 kDa and 26.3 kDa, respectively). Ultracentrifugation analysis revealed that DacA_1–287_ corresponded to a stable tetramer with an estimated molecular mass of 150 kDa (Fig. 6C). DacA_140–358_ was detected with an estimated molecular mass of 42 kDa (Fig. 6C), which is between a monomer and a dimer. Similar results, for either DacA_1–287_ or DacA_140–358_, were obtained using gel filtration (data not shown). To further determine the oligomerization of DacA_140–358_, we treated DacA_140–358_ with glutaraldehyde and then analyzed by Western blot with the anti-DacA antibody. A dominant dimer-sized band was detected in the glutaraldehyde-treated sample, whereas the untreated sample was detected exclusively as a monomer (Fig. 6D). Therefore, DacA_140–358_ is stable as a dimer at the native condition.

According to the structural study of DisA and the sequence similarity between DacA and DisA [22], ATP might interact with DacA at the N-terminal domain. In this study, we analyzed the ATP binding by DacA using a gel mobility shift assay. In this assay, no divalent ion was provided, thus no c-di-AMP could be formed from ATP. In the absence of ATP, both DacA and DacA_1–287_ showed multiple bands in a native gel. However, with the presence of ATP in the protein samples, all the proteins migrated as a single band. Additionally, DacA migrated faster in the presence of ATP than in the absence of ATP (Fig. 6E), suggesting that ATP interacts with DacA and likely alters DacA's conformation. A similar result was observed with DacA_1–287_, but not DacA_140–358_ (Fig. 6E), indicating that the N-terminal domain of DacA is responsible for ATP binding.

To test whether the truncated proteins still have diadenylate cyclase activity, these proteins were incubated with ATP followed by HPLC analysis. When 2.5 µM protein was used, no enzymatic activity was detected with either protein (data not shown). At 10 µM protein, DacA_1–287_ showed weaker activities than that of DacA, while DacA_140–358_ did not show any enzymatic activity (Fig. 6F). These data suggest that both the N-terminal and the C-terminal domains of DacA are required for DacA's activities. However, the catalytic domain is located at the N-terminus of the protein.

### RHR motif is essential for ATP binding and the diadenylate cyclase activity

**Figure 7:**
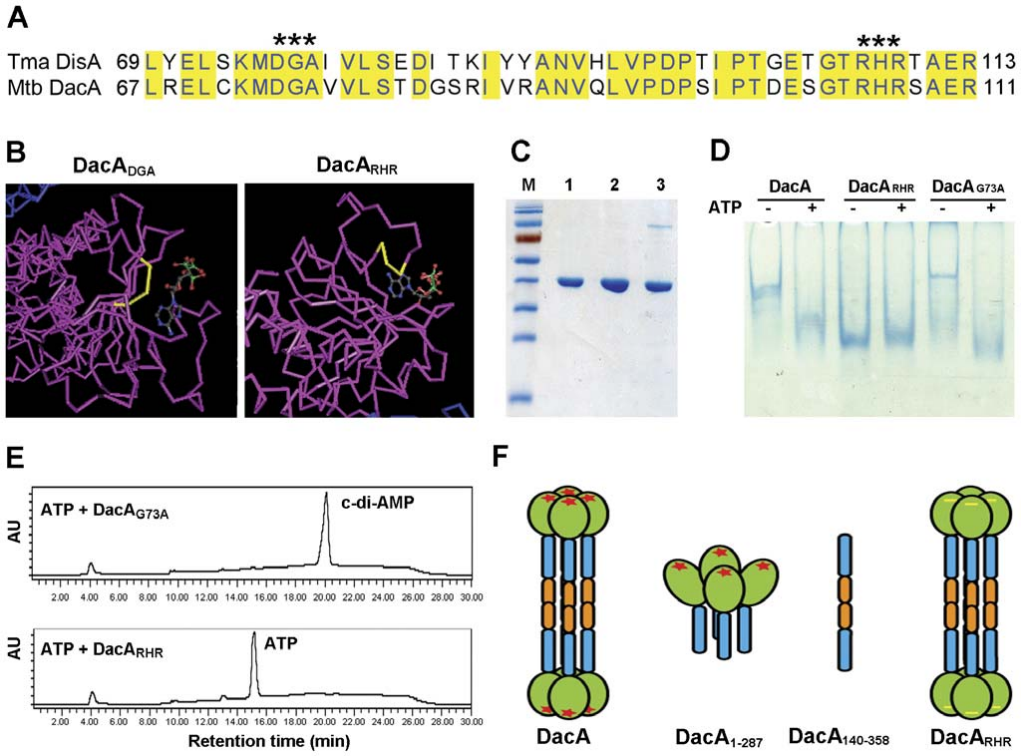
Function of the DGA and RHR motifs in DacA. (A) Partial sequence alignment of *M. tuberculosis* DacA and *T maritima* DisA showing the conserved DGA and RHR motifs. Identical residues between the two proteins are highlighted in yellow blocks. (B) Potential contact of DGA and RHR motifs with ATP generated from *T. maritima* DisA [22] using the Cn3D software. The three amino acids highlighted in yellow represent either DGA or RHR, as indicated. (C) SDS-PAGE of purified DacA, DacA_RHR_ and DacA_G73A_. Lane M, MW marker; lanes 1–3 are purified DacA, DacA_RHR_ and DacA_G73A_, respectively. (D) ATP binding by DacA_RHR_ and DacA_G73A_. Proteins either in the presence (+) or absence (−) of ATP were separated by electrophoresis with a native gel and stained with Coomassie Brilliant Blue. (E) Analysis of diadenylate cyclase activity of 10 µM DacA_G73A_ and DacA_RHR_ in the presence of ATP using HPLC. (F) Structural modeling of DacA and its derivative polypeptides. Three domains of DacA from the N-terminus to the C-terminus are colored in green, blue and orange, respectively. A red star represents one molecule of ATP. A yellow line in DacA_RHR_ indicates the mutation of RHR motif.

It has been shown structurally that the functional motifs (DGA and RHR) of DisA interact with c-di-AMP [22]. These motifs are also conserved in DacA (Fig. 7A). According to structural modeling using the DisA protein of *T. maritima*, these motifs may be in contact with ATP (Fig. 7B). In this study, we substituted the residuals within DGA and RHR motifs of DacA to determine the function of these motifs in the ATP binding and the diadenylate cyclase activity. Mutation of DGA to AAL (DacA_DGA_) or AAA (DacA_DG_) resulted in expression within inclusion bodies. Structural studies with DisA showed that the glycine in the DGA motif that interacts with c-di-AMP [22]. Therefore, we mutated this glycine to alanine (DacA_G73A_) and RHR to AAA (DacA_RHR_). We purified DacA_G73A_ and DacA_RHR_ to high homogeneity; the protein showed the same apparent molecular weight as the native DacA (Fig. 7C). The interaction of ATP with these two proteins was analyzed using gel mobility shift assay. The result showed that the mobility of DacA_G73A_ was identical to the native DacA either in the presence or absence of ATP. In contrast, the mobility of DacA_RHR_ was not shifted in the presence of ATP (Fig. 7D), indicating that the RHR motif is important in the interaction with ATP. The diadenylate cyclase activity of DacA_G73A_ and DacA_RHR_ was also determined using HPLC. The result showed that mutation of RHR in DacA completely abolished the production of c-di-AMP (Fig. 7E), whereas DacA_G73A_ retained the diadenylate cyclase activity, suggesting that RHR, but not the glycine in DGA, is essential for DacA's diadenylate cyclase activity.

## Discussion

In this study, we identified *M. tuberculosis* DacA as a diadenylate cyclase similar to the *B. subtilis* DisA protein, as predicted [22]. DacA also exhibited residual ATPase and ADPase activities, which have not been described with the DisA proteins of *B. subtilis* and *T. maritima* [22]. There may be several possible explanations for the ATPase and the ADPase activities of DacA. When one molecule of c-di-AMP is synthesized from two ATP molecules, β- and γ-phosphates are eventually removed, and only α-phosphate remains in c-di-AMP (Fig. 4B). Hydrolysis of β- and γ-phosphate by DacA could be catalyzed either by the specific structure of ATPase and ADPase, or more likely as a part of the diadenylate cyclase activity. Thus, ADP might be an intermediate product, which can be utilized by DacA to produce c-di-AMP. Furthermore, the ATPase and the ADPase activities are unlikely due to contaminations in the purified DacA. Our data clearly showed that, with higher concentration of DacA, the yields of ADP and AMP became lower (Fig. 3D), rather than proportionally higher, as would be expected if the activity was due to contaminations in the DacA preparation. Additionally, GTPase activity has been previously observed in a GGDEF domain protein [38], and an atypical GGDEF domain of YybT exhibited unexpected ATPase activity as well [16].

Many diguanylate cyclases also have a phosphodiesterase domain, which converts c-di-GMP to pGpG. However, a phosphodiesterase domain was not predicted from the DisA structural study, whereas *B. subtilis* YybT protein functions partly as c-di-AMP phosphodiesterase [16]. In our reactions with DisA and DacA, we detected several nucleotides in addition to c-di-AMP that were generated from ATP. In particular, the presence of AMP and trace amount of pApA led us to determine whether DacA also has a phosphodiesterase activity. By providing c-di-AMP in the reaction, we demonstrated that DacA was unable to hydrolyze c-di-AMP. Therefore, DacA does not have c-di-AMP phosphodiesterase activity. The AMP and pApA products that we detected in the reactions might be intermediates formed during synthesis of c-di-AMP. In addition, *M. tuberculosis* Rv0805 has been reported as a phosphodiesterase of certain cyclic nucleotides [40], [41]. In this study, we also incubated Rv0805 protein with c-di-AMP and analyzed the products using TLC. Our preliminary study showed that this protein could not hydrolyze c-di-AMP either (data not shown). Therefore, the phosphodiesterase of c-di-AMP in *M. tuberculosis* remains unknown.

DisA forms a large octamer, and each monomer contains three domains including an N-terminal catalytic domain and a C-terminal HhH domain [22]. The structural study of DisA has shown that two tetramers interact via domain 1. The main interaction for a four-fold symmetry is mediated by domain 2, but also includes domains 1 and 3 [22]. In the present study, DacA was purified as an octamer, which is comparable to the oligomerization of DisA. Deletion of the C-terminal domain of DacA revealed a tetramer, while deletion of the N-terminal domain of DacA exhibited a dimer. These data suggest that in the oligomerization of DacA, the N-terminal domain contributes to tetramerization, and the C-terminal HhH domain is responsible for additional dimerization. This model deviates from the DisA model that has been reported [22], possibly because DacA differs from DisA.

According to the sequence alignment with DisA and our mutagenesis results of DacA, the catalytic moiety of DacA is located at the N-terminal domain. Furthermore, the RHR motif is essential for DacA's ATP-binding and the enzymatic activity. Deletion of the N-terminal catalytic domain of DacA completely abolishes DacA's ATP binding and the enzymatic activity. Surprisingly, the activities of DacA are dramatically reduced by deletion of the HhH domain, while the tetrameric catalytic domains and the ATP binding ability are still retained. This suggests that the enzymatically active conformation is required, and that the HhH domain plays a role in stabilizing the active conformation. Therefore, we have proposed a structural model of DacA based on our results (Fig. 7F). In this model, we hypothesize that the removal of the C-terminal domain results in a conformational alteration, which significantly reduces the diadenylate cyclase activity, but retains the capability to bind ATP. On the other hand, deletion of the N-terminal domain or mutation of the RHR motif abolishes the ATP-binding and the diadenylate cyclase activity.

The activities of DacA are strictly dependent on divalent metal ions. Generally, the catalytic activities are exhibited in the presence of Mg^2+^, Mn^2+^ or Co^2+^. These divalent ions are co-factors for enzymes, such as adenylate cyclases and phosphodiesterases [16], [42]. In *M. tuberculosis*, Mg^2+^ or Mn^2+^ is needed for the activity of Cya (Rv1625c), an adenylate cyclase [42]. We have also detected that Rv0805 has a preference for Mn^2+^ or Co^2+^ (Bai and McDonough, manuscript submitted). These results suggest that such cations play important roles in bacterial signaling. Furthermore, similar to several enzymes, such as YybT and Rv0805 [16], the diadenylate cyclase activity of DacA is more active at a basic pH, rather than at an acidic or a neutral condition. The biological equivalence of the pH effect warrants further investigation.

The roles of c-di-GMP in bacterial pathogenesis have been well established. However, little is known about the function of c-di-AMP. Our future studies will explore the role of c-di-AMP in the biology and pathogenesis of *M. tuberculosis*.
